# Multimodal Diagnostic Cross-Check: A Correlative Study of Fine-Needle Aspiration Cytology, Ultrasonography, Thyroid Profile, and Histopathology in Thyroid Swellings

**DOI:** 10.7759/cureus.113274

**Published:** 2026-07-24

**Authors:** Farheen Khan, Nishi Tandon, Yoshita Agnihotri, Suboohi Khanam, Andleeb Zehra, Nirupma Lal

**Affiliations:** 1 Pathology, Era's Lucknow Medical College and Hospital, Lucknow, IND

**Keywords:** bethesda system, diagnostic accuracy, fine-needle aspiration cytology, histopathology, thyroid function tests, thyroid nodule, tirads, ultrasonography

## Abstract

Background: Thyroid swellings are common in surgical and endocrine practice, and accurate preoperative discrimination of benign from malignant nodules guides surgical decision-making and avoids unnecessary intervention. Preoperative work-up typically combines fine-needle aspiration cytology (FNAC), ultrasonography (USG) with Thyroid Imaging Reporting and Data System (TI-RADS) scoring, and serum thyroid-stimulating hormone (TSH) measurement, with histopathological examination (HPE) as the reference standard.

Objectives: To evaluate the diagnostic performance and correlation of FNAC (Bethesda System), the American College of Radiology (ACR) TI-RADS USG, and serum TSH status against histopathological examination as the reference standard.

Methods: In this single-center, prospective, cross-sectional study conducted from June 2024 to May 2025, 178 consecutive patients underwent clinical evaluation, serum TSH measurement, USG using ACR TI-RADS, and FNAC, with results reported according to the Bethesda System. Sixty-eight patients (38.20%) underwent surgery, with HPE as the reference standard. Diagnostic performance estimates were calculated only in this surgically verified subgroup, with 95% Wilson score confidence intervals (CIs).

Results: The mean age was 44.88 ± 15.34 years. Of the 178 patients, 108 (60.67%) were female (female-to-male ratio, 1.54:1). Hypothyroidism was the most common thyroid functional state, occurring in 103 (57.87%) patients. Among the 68 operated patients, malignant or borderline histopathology, including non-invasive follicular thyroid neoplasm with papillary-like nuclear features (NIFTP), was confirmed in 36 (52.94%) patients. FNAC showed sensitivity of 58.33%, specificity of 100.00%, positive predictive value (PPV) of 100.00%, negative predictive value (NPV) of 68.09%, and accuracy of 77.94% (κ = 0.57). USG showed sensitivity of 75.00%, specificity of 78.12%, PPV of 79.41%, NPV of 73.53%, and accuracy of 76.47% (κ = 0.53). The Bethesda category correlated strongly with HPE (χ² = 50.43, *P* < 0.0001); thyroid functional status did not (*P* = 0.309).

Conclusions: Within the surgically verified subgroup, FNAC and USG TI-RADS provided complementary diagnostic information. FNAC showed high specificity and PPV but limited sensitivity, whereas USG TI-RADS showed higher sensitivity with lower specificity. Interpretation is limited by partial histopathological verification.

## Introduction

Thyroid swellings range from benign colloid goiter and inflammatory thyroiditis to malignant neoplasms and are among the most common endocrine disorders encountered clinically. Palpable nodules are identified in only a modest proportion of adults, but high-resolution ultrasonography (USG) detects subclinical nodules far more often, particularly in women and older individuals. Thyroid carcinoma is the leading endocrine malignancy worldwide, and its recorded incidence has risen substantially in recent decades, a trend driven in part by wider use of high-resolution imaging rather than a true increase in disease burden alone [1].

The core diagnostic problem in nodular thyroid disease is separating the small fraction of clinically important malignant nodules from a much larger benign population so that unnecessary thyroidectomy is avoided while malignant disease is still caught and treated promptly. Preoperative evaluation has accordingly become a multimodal pathway combining clinical assessment, thyroid function testing, USG with standardized risk scoring, and cytopathological sampling by fine-needle aspiration [2].

Among available preoperative tests, fine-needle aspiration cytology (FNAC) is favored for its low cost, minimal invasiveness, and extensive prior validation. Cytological findings are reported using the Bethesda System for Reporting Thyroid Cytopathology, which assigns each specimen to one of the six categories, each linked to an estimated malignancy risk that guides subsequent management [3]. Reported sensitivity and specificity of FNAC vary across published series, reflecting differences in operator experience, sampling technique, and case mix, particularly the proportion of follicular-patterned lesions [4,5].

USG provides a complementary structural assessment, most often standardized through the American College of Radiology (ACR) Thyroid Imaging Reporting and Data System (TI-RADS), in which nodules are scored based on five sonographic features - composition, echogenicity, shape, margin, and echogenic foci - to yield a final category ranging from TR1 to TR5 [6]. Serum thyroid-stimulating hormone (TSH) is routinely measured as part of initial evaluation; a lower serum TSH has been loosely associated with higher malignancy risk in some epidemiological data, though this relationship is inconsistent enough that TSH alone cannot reliably discriminate malignancy in an individual patient [7]. Definitive diagnosis still rests on the histopathological assessment of resected tissue, now classified according to the 2022 World Health Organization (WHO) Classification of Thyroid Neoplasms, which reorganized follicular-patterned lesions and recognized non-invasive follicular thyroid neoplasm with papillary-like nuclear features (NIFTP) as a distinct borderline entity [8].

FNAC, USG TI-RADS, and thyroid function testing have each been extensively studied in isolation, but correlative studies evaluating all three together against histopathology in one cohort remain comparatively scarce, particularly in the Indian subcontinent. This study addresses that gap. Its primary aim was to examine the multimodal diagnostic correlation among FNAC using the Bethesda System, USG using ACR TI-RADS, serum TSH status, and histopathology. Its secondary aims were to quantify the individual diagnostic performance of FNAC and USG and their inter-modality agreement with HPE.

The study objectives are as follows:

Primary objective: To evaluate the diagnostic performance and correlation of FNAC using the Bethesda System, USG using ACR TI-RADS, and serum TSH status against histopathological examination as the reference standard.

Secondary objectives: To quantify the individual diagnostic performance of FNAC and USG (ACR TI-RADS) and assess their agreement with histopathological findings.

## Materials and methods

Study design and ethical approval

This was a single-center, prospective, cross-sectional, observational correlative study conducted from June 2024 to May 2025 at Era’s Lucknow Medical College and Hospital, Era University, Lucknow, India. This study was approved by the Institutional Ethics Committee, Era’s Lucknow Medical College and Hospital, Era University, Lucknow (IEC Registration No. ECR/717/Inst./UP/2015/RR-21; approval reference ELMC&H/R.Cell/2024-A/208; approved on May 18, 2024), and was conducted in accordance with the Declaration of Helsinki. Written informed consent was obtained from every participant, and confidentiality was maintained throughout.

Study population and sampling

**Figure 1:**
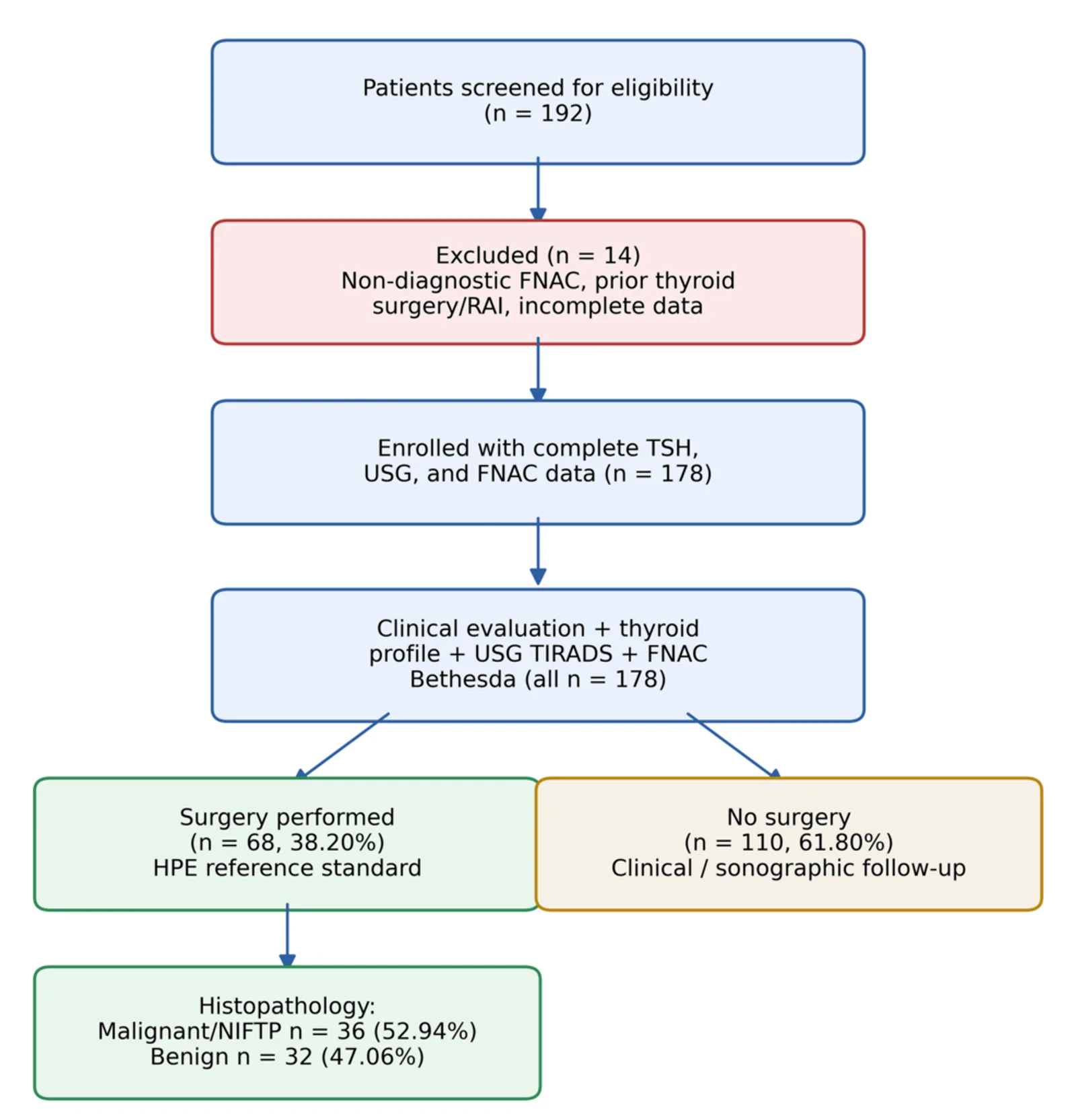
Flow diagram of patient enrollment, index testing, and histopathological verification. The image was created by Farheen Khan and Nishi Tandon using Microsoft Paint (Microsoft Corporation, Redmond, WA). FNAC, fine-needle aspiration cytology; HPE, histopathological examination; TSH, thyroid-stimulating hormone; USG, ultrasonography; NIFTP, non-invasive follicular thyroid neoplasm with papillary-like nuclear features

Consecutive patients with clinically palpable or incidentally imaging-detected thyroid swellings were screened for eligibility. Of the 192 patients screened, 178 met the inclusion criteria and were enrolled: age >16 years, palpable or imaging-detected swelling, willingness to provide consent, and complete TSH, USG, and FNAC data. Patients with prior radioactive iodine therapy or neck irradiation, previous thyroid surgery, a non-diagnostic aspirate (Bethesda category I), refusal of consent, or incomplete data were excluded. Of the 192 screened patients, 14 were excluded according to the prespecified eligibility criteria. A detailed flow diagram is shown in Figure 1.

Unit of analysis and index nodule selection

The unit of analysis was the patient. In patients with a solitary thyroid nodule, the same nodule was assessed by ultrasonography, FNAC, and histopathology. In patients with multinodular thyroid disease, the index nodule was defined as the nodule with the highest ACR TI-RADS category or, when categories were similar, the largest nodule that underwent FNAC and guided the surgical decision. Cytological, ultrasonographic, and histopathological findings were matched using lesion location, size, and operative/pathology records.

Thyroid function assessment

Serum TSH was measured in all patients using an Abbott Alinity i chemiluminescent immunoassay analyzer (Abbott Diagnostics, Abbott Park, IL; reference range 0.4-4.5 mIU/L) according to the manufacturer’s instructions. Patients were classified as euthyroid, hypothyroid (TSH >4.5 mIU/L), or hyperthyroid (TSH <0.4 mIU/L).

Ultrasonographic evaluation

A single experienced radiologist performed high-resolution B-mode ultrasonography using a GE LOGIQ P9 ultrasound system (GE Healthcare, Chicago, IL, USA) equipped with a 7.5-12 MHz linear-array transducer to limit interobserver variability. Nodules were scored according to ACR TI-RADS across composition, echogenicity, shape, margin, and echogenic foci, yielding a category from TR1 through TR5 [6].

Cytological evaluation

FNAC was performed by an experienced cytopathologist using a 23-25 gauge needle, with ultrasound guidance for complex or cystic lesions. Smears were stained using Papanicolaou and May-Grünwald-Giemsa methods and classified according to the Bethesda System, categories II through VI; category I specimens were excluded per protocol [3]. The radiologist was blinded to cytological findings at the time of ultrasonographic interpretation. The cytopathologist was blinded to the ACR TI-RADS category. Histopathological assessment was performed as part of routine clinical care; complete blinding to FNAC and TI-RADS findings was not ensured.

Histopathological evaluation

Of the 178 patients, 68 (38.20%) underwent surgery. Specimens were fixed in 10% neutral buffered formalin, routinely processed, paraffin-embedded, sectioned at 3-5 µm, and stained with hematoxylin and eosin. Immunohistochemistry was performed where required to establish the final diagnosis. Diagnoses followed the 2022 WHO Classification [8]. For the primary diagnostic performance analysis, histopathological outcomes were classified as benign versus malignant/borderline, with NIFTP included in the malignant/borderline group because it is a diagnostically challenging neoplasm that commonly requires surgical excision for definitive classification.

Statistical analysis

Analyses were performed in Python (pandas, NumPy, SciPy, scikit-learn). Bethesda V/VI and TI-RADS 4/5 were defined as positive for malignancy in the 68 surgically confirmed cases. The reference outcome was defined as malignant/borderline histopathology, with NIFTP included in this group for the primary analysis. Sensitivity, specificity, PPV, NPV, and accuracy were calculated against HPE, with 95% CIs calculated using the Wilson score method [9]. Categorical associations were tested using the chi-square test or Fisher’s exact test, as appropriate. Intermodality agreement was quantified using Cohen’s kappa (κ): 0.01-0.20, slight; 0.21-0.40, fair; 0.41-0.60, moderate; 0.61-0.80, substantial; and 0.81-1.00, almost perfect [10]. Significance was set at p < 0.05. Diagnostic performance was evaluated in the surgically verified subgroup only. For the primary FNAC analysis, Bethesda categories II, III, and IV were classified as negative, and Bethesda categories V and VI were classified as positive. As surgery was performed according to routine clinical indications rather than the study protocol, the estimates represent test performance among operated patients and should not be interpreted as population-level estimates for all patients presenting with thyroid nodules.

## Results

Demographic and functional profile

The mean age was 44.88 ± 15.34 years (median, 44.0; range, 16-75 years), with the highest concentration of patients in the fourth and fifth decades. Of the 178 enrolled patients, 108 (60.67%) were female and 70 (39.33%) were male (F:M = 1.54:1). Hypothyroidism was the most common functional state, observed in 103 (57.87%) patients, followed by euthyroidism in 70 (39.33%) patients and hyperthyroidism in 5 (2.81%) patients. The mean TSH level was 4.95 ± 2.71 mIU/L and did not differ significantly between HPE-malignant cases (5.11 ± 2.85 mIU/L) and HPE-benign cases (4.53 ± 2.35 mIU/L; p = 0.36). Demographic and functional data are summarized in Table 1.

**Table 1:** Baseline demographic and thyroid functional characteristics of enrolled patients (n = 178). Values are presented as *n* (%) unless otherwise stated. The mean age was 44.88 ± 15.34 years, and the mean serum TSH was 4.95 ± 2.71 mIU/L. TSH, thyroid-stimulating hormone

Parameter	Category	*n* (%)
Age group (years)	≤40	76 (42.69)
	41-60	71 (39.89)
	> 60	31 (17.42)
Sex	Female	108 (60.67)
	Male	70 (39.33)
Thyroid status	Euthyroid	70 (39.33)
	Hypothyroid	103 (57.87)
	Hyperthyroid	5 (2.81)

Sonographic and cytological distribution

TI-RADS 2 was the most prevalent sonographic category, observed in 87 (48.88%) patients, while TI-RADS 4/5 categories were present in 52 (29.21%) patients. Bethesda II predominated on cytology and was observed in 143 (80.34%) patients, whereas Bethesda V/VI categories comprised 21 (11.79%) patients. The four Bethesda III and 10 Bethesda IV cases represented the diagnostically indeterminate subset in whom sonographic correlation was most valuable. The full distribution is presented in Table 2.

**Table 2:** Distribution of ACR TI-RADS and Bethesda cytology categories among enrolled patients (n = 178). TI-RADS 4/5 categories were present in 52 (29.21%) patients, and Bethesda V/VI categories were present in 21 (11.79%) patients. TI-RADS 4/5 categories were considered test-positive for the primary ultrasonographic diagnostic performance analysis. Bethesda V/VI categories were considered test-positive for the primary FNAC analysis. TI-RADS, Thyroid Imaging Reporting and Data System; FNAC, fine-needle aspiration cytology; ACR, American College of Radiology

TI-RADS	*n* (%)	Bethesda	*n* (%)
TR1	1 (0.56)	II	143 (80.34)
TR2	87 (48.88)	III	4 (2.25)
TR3	38 (21.35)	IV	10 (5.62)
TR4	23 (12.92)	V	19 (10.67)
TR5	29 (16.29)	VI	2 (1.12)

Histopathological spectrum

**Figure 2:**
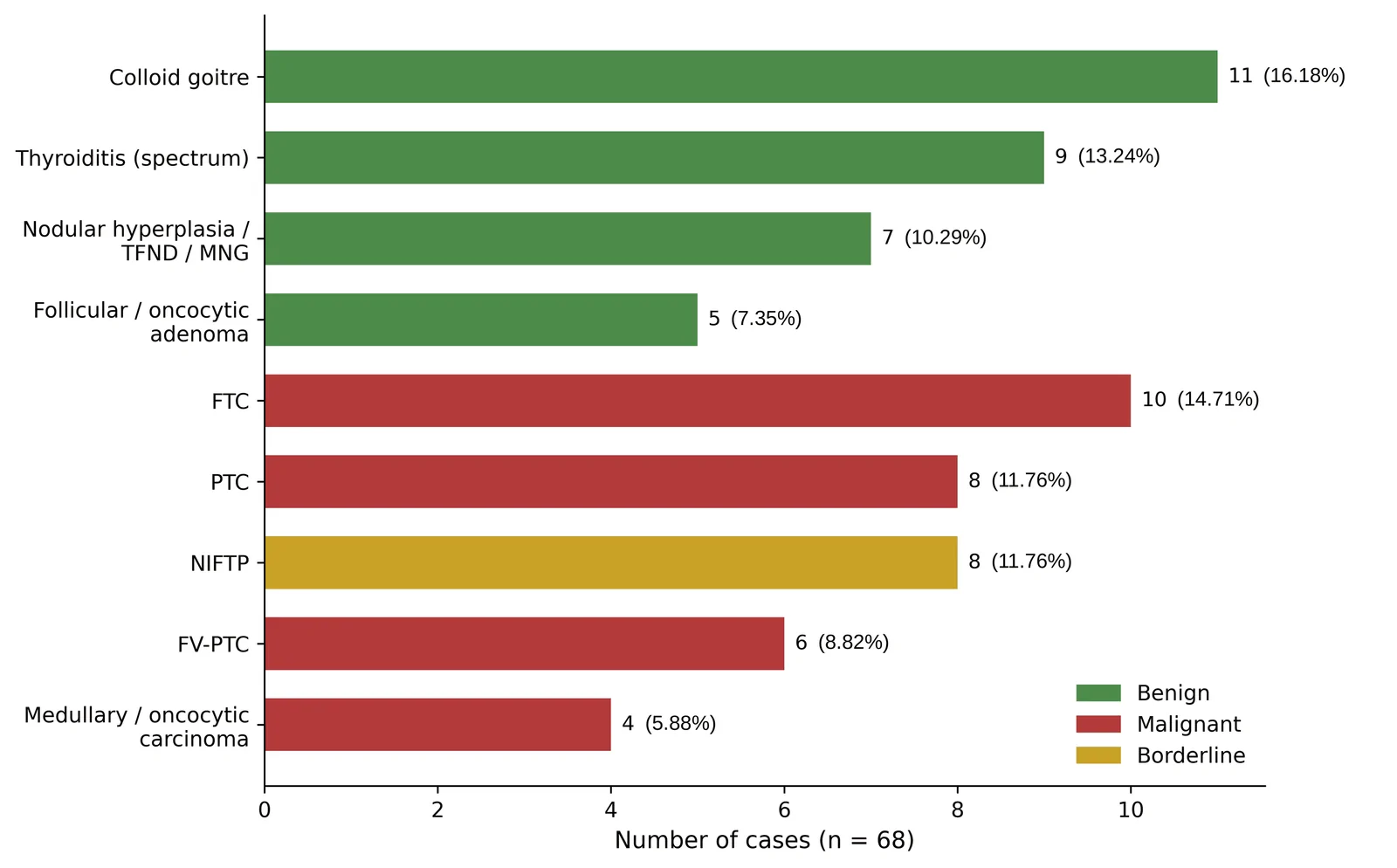
Distribution of histopathological diagnoses among surgically verified patients (n = 68) FTC, follicular thyroid carcinoma; PTC, papillary thyroid carcinoma; FV-PTC, follicular-variant papillary thyroid carcinoma; NIFTP, non-invasive follicular thyroid neoplasm with papillary-like nuclear features; TFND, thyroid follicular nodular disease; MNG, multinodular goiter

Of the 178 enrolled patients, 68 (38.20%) underwent surgery and had histopathological confirmation, while the remaining 110 (61.80%) did not undergo surgery during the study period because they were managed conservatively or monitored with clinical and ultrasonographic surveillance according to routine clinical decision-making. Among the 68 operated patients, malignant/borderline histopathology was identified in 36 (52.94%) patients, including 8 (11.76%) patients with NIFTP, whereas benign disease was identified in 32 (47.06%) patients. The malignant/borderline histopathology prevalence of 36 out of 68 (52.94%) reflects the surgically verified subgroup and should not be interpreted as the prevalence among all 178 enrolled patients, because surgical referral was based on clinical decision-making and likely selected patients with higher-risk findings. FTC was the most frequent malignant diagnosis, observed in 10 (14.71%) patients, followed by classical papillary thyroid carcinoma (PTC) in 8 (11.76%) patients, NIFTP in 8 (11.76%) patients, and follicular-variant PTC in 6 (8.82%) patients. Colloid goiter was the most frequent benign diagnosis, observed in 11 (16.18%) patients. The full breakdown is shown in Figure 2.

Correlation with histopathology

**Figure 3:**
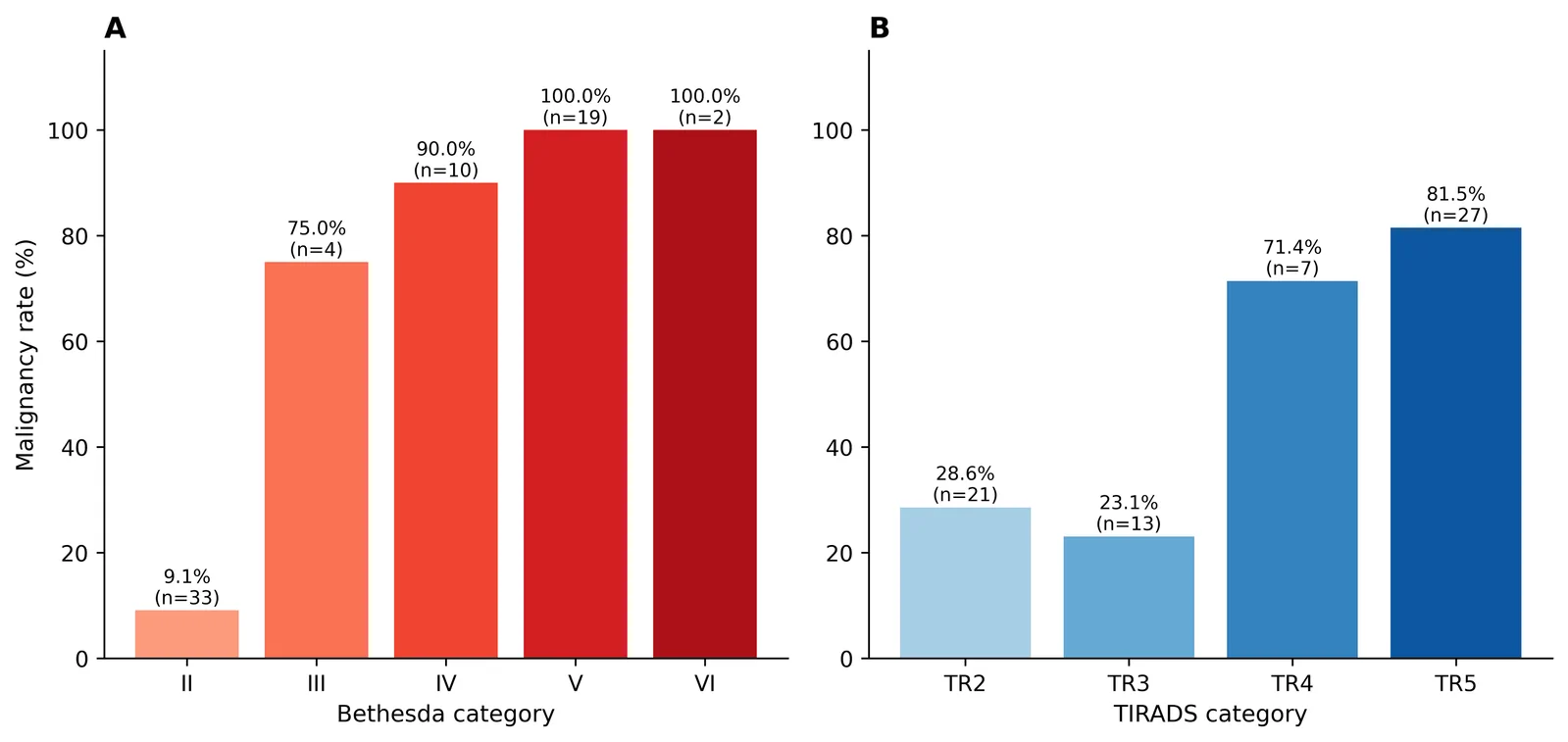
Malignant/borderline histopathology rates according to Bethesda cytology category and ACR TI-RADS category in surgically verified patients (n = 68). (A) Proportion of malignant/borderline histopathology across Bethesda categories II to VI. (B) Proportion of malignant/borderline histopathology across ACR TI-RADS categories TR2 to TR5. HPE, histopathological examination; TI-RADS, Thyroid Imaging Reporting and Data System

Bethesda category was strongly associated with HPE (χ² = 50.43, *P* < 0.0001), with concordance strongest at both extremes: all Bethesda V/VI cases had malignant/borderline histopathology, while most Bethesda II cases were correctly benign, except for three unexpected malignancies (predominantly FTC and NIFTP). TI-RADS also correlated significantly with malignancy (χ² = 19.45, *P* = 0.0002), increasing stepwise from TR2 to TR5; malignant/borderline histopathology was identified in 9 of 34 (26.47%) patients with combined TI-RADS 2/3 nodules. Thyroid functional status showed no such association (χ² = 2.35, *P* = 0.309). Category-wise malignant/borderline histopathology rates are illustrated in Figure 3.

Diagnostic performance

FNAC showed high specificity and PPV, with no false-positive results at the Bethesda V/VI positivity threshold. However, 15 malignant/borderline lesions were classified below this threshold, resulting in limited sensitivity. The 15 false-negative FNAC results occurred predominantly in follicular-patterned lesions, including FTC, NIFTP, and follicular-variant PTC; three were classified as Bethesda II. USG achieved higher sensitivity than FNAC, reflecting its ability to flag structural atypia even when FNAC is cytologically reassuring or indeterminate, at the cost of lower specificity: its seven false positives reflected recognized sonographic mimics of malignancy. Both modalities showed similar, moderate kappa agreement with HPE, but the differing pattern of error each makes is the practical rationale for combining them. Because PPV, NPV, and overall accuracy are influenced by disease prevalence and were calculated only in the surgically verified subgroup, these estimates may not be directly generalizable to unselected patients with thyroid nodules. The observed diagnostic performance should therefore be interpreted in the context of partial reference-standard verification. Full diagnostic performance metrics with 95% CIs are presented in Table 3.

**Table 3:** Diagnostic performance of FNAC and ACR TI-RADS against malignant/borderline histopathology in surgically verified patients (n = 68) Values are presented as percentages with 95% Wilson score confidence intervals. FNAC positivity was defined as Bethesda categories V and VI, and ultrasonographic positivity was defined as ACR TI-RADS categories 4 and 5. PPV, positive predictive value; NPV, negative predictive value; ACR, American College of Radiology; FNAC, fine-needle aspiration cytology; TI-RADS, Thyroid Imaging Reporting and Data System; USG, ultrasonography

Parameter	FNAC (Bethesda)	USG (TI-RADS)
Sensitivity	58.33% (42.20-72.86)	75.00% (58.93-86.25)
Specificity	100.00% (89.28-100.00)	78.12% (61.24-88.98)
PPV	100.00% (84.54-100.00)	79.41% (63.20-89.65)
NPV	68.09% (53.83-79.60)	73.53% (56.88-85.40)
Accuracy	77.94% (66.74-86.15)	76.47% (65.14-84.97)
Cohen's kappa	0.57 (moderate)	0.53 (moderate)

## Discussion

This prospective study evaluated 178 patients with thyroid swellings through a structured multimodal pathway, with histopathological correlation in 68 operated cases. The findings are relevant across three domains: cohort epidemiology, pathological spectrum, and the comparative performance of FNAC and USG TI-RADS against histopathology.

The age distribution and pronounced female predominance (F:M = 1.54:1) mirror large population-based series [1,2]. Although hypothyroidism was the most common functional abnormality, thyroid functional status showed no association with malignancy, and mean TSH did not differ between benign and malignant groups, consistent with the broader view that TSH, while essential to overall management, lacks the discriminatory power to be used alone in operative decision-making [7]. Practically, this means thyroid function testing should continue to inform management, but biopsy and surgical decisions should rest on cytological and sonographic risk stratification rather than biochemical status.

USG TI-RADS achieved a sensitivity of 75.00%, specificity of 78.12%, and accuracy of 76.47%. The malignant/borderline histopathology rate increased progressively across TI-RADS categories and reached 22 of 27 (81.48%) among TR5 nodules. This graded pattern is consistent with the behavior expected of a well-calibrated risk stratification system and with contemporary ACR TI-RADS validation data [6]. Its false positives, comprising complex adenomatous goiter, densely calcified nodules, and Hashimoto pseudonodules, are well-documented sonographic mimics of malignancy [11]. This suggests that radiologists should apply particular caution when scoring nodules in a heterogeneous or pseudonodular gland, especially where autoimmune thyroiditis is common.

In this surgically verified cohort, Bethesda V/VI cytology showed 100% specificity and PPV, although the confidence intervals were wide; these findings support its high-risk clinical significance but require cautious interpretation [3]. Its more modest sensitivity (58.33%) is the more clinically important limitation, explained largely by the pathological spectrum encountered: follicular-patterned lesions such as FTC and follicular-variant PTC are poorly discriminated by cytology because their defining feature, capsular or vascular invasion, is an architectural finding visible only on histological sections [4]. NIFTP poses a similar diagnostic problem, since it may show cytological features overlapping with follicular-patterned lesions. In the present study, NIFTP was included within the malignant/borderline reference outcome because it is a clinically relevant lesion requiring definitive histopathological classification; this outcome definition should be considered when interpreting diagnostic-performance estimates [5,8]. These two entities, together with the three unexpected Bethesda II malignancies, account for most of the false negatives observed.

Malignant/borderline histopathology was identified in 9 of 10 (90.00%) Bethesda IV cases. Several factors plausibly contribute: tertiary referral centers accumulate a case mix enriched for complex follicular neoplasms already triaged away from primary care. The inclusion of NIFTP within the malignant/borderline outcome category may have increased the observed rate of clinically significant histopathology [8]. The small absolute number of Bethesda IV cases here makes the proportion inherently less stable. The high observed malignant/borderline histopathology rate among Bethesda IV lesions warrants confirmation in larger cohorts and should be interpreted alongside ultrasound findings, patient factors, and available molecular testing.

FTC outnumbering classical PTC as the leading malignancy represents a departure from most published Indian and international series, where PTC usually predominates. This may reflect regional iodine nutritional status, which is known to influence the follicular-to-papillary carcinoma ratio, together with a referral pattern favoring diagnostically ambiguous follicular neoplasms [1]. Whatever the explanation, it reinforces the broader theme of this study: follicular-patterned disease represents the principal blind spot of cytology-based screening.

The central, actionable finding is the complementary behavior of FNAC and USG TI-RADS: FNAC's high specificity and PPV make it the preferred tool for confirming malignancy, while USG's higher sensitivity makes it more useful for flagging structural concern in cytologically reassuring or indeterminate lesions [3,6]. The differing sensitivity-specificity profiles suggest that FNAC and USG may provide complementary information; however, the diagnostic performance of a prespecified combined testing strategy was not evaluated in this study. Molecular adjuncts such as BRAF, RAS family, and TERT promoter testing represent a promising next step for refining risk stratification specifically within indeterminate Bethesda III/IV categories [3,12].

Limitations

As a single tertiary-center study, this cohort is subject to referral bias toward complex, higher-risk lesions. Verification bias is a major limitation because histopathological confirmation was available for only 68 of 178 enrolled patients (38.20%), as surgery was undertaken according to routine clinical indications rather than a study-mandated protocol. The surgically verified subgroup was therefore likely enriched for patients with suspicious clinical, cytological, or sonographic findings, as reflected by the malignant/borderline histopathology prevalence of 36 of 68 (52.94%) participants. Consequently, PPV, NPV, overall accuracy, and observed disease prevalence should be interpreted as estimates for the operated subgroup rather than for the full cohort or for unselected patients with thyroid nodules. Occult malignancies among unoperated patients could not be excluded.

FNAC’s inherent limitation in follicular-patterned neoplasms, where the adenoma-carcinoma distinction depends on histological features unavailable to aspiration cytology, reflects a constraint of the technique itself rather than a limitation of this study; however, it materially affects the sensitivity reported here. A complete thyroid profile, including T3 and T4, was not available for the entire cohort. NIFTP was included within the malignant/borderline outcome category because of its diagnostic and management relevance; this classification choice may have influenced the reported diagnostic performance estimates. Next, histopathologists were not fully blinded to prior clinical or imaging findings because histopathological assessment was performed as part of routine clinical practice, which may have introduced interpretation bias. Finally, because ultrasonographic examinations were interpreted by a single radiologist, inter-observer reproducibility could not be assessed. Residual variability in cytological and radiological interpretation cannot be excluded, and the single-center design may limit generalizability

## Conclusions

This study found that FNAC and USG TI-RADS provided differing and potentially complementary preoperative information. In this surgically verified cohort, Bethesda V/VI FNAC showed 100% specificity and PPV, although these estimates had wide confidence intervals; its limited sensitivity reflected the recognized cytological challenges of follicular-patterned neoplasms and NIFTP. USG TI-RADS offered higher sensitivity with somewhat lower specificity, and both modalities showed moderate agreement with histopathology; thyroid functional status showed no independent association with malignancy. Follicular-patterned lesions, including FTC and NIFTP, were the most diagnostically challenging entities overall. Within the surgically verified subgroup, these findings support the complementary use of FNAC and USG TI-RADS in preoperative assessment. Larger prospective studies with more complete reference-standard verification are needed before the reported diagnostic-performance estimates can be generalized to all patients with thyroid nodules.
